# An NMR-Based Protocol for Profiling the Endo- and Exo-Metabolomes in Aβ_1-42_ Treated Human Astrocytes from Healthy and Alzheimer’s Disease Donors

**DOI:** 10.3390/metabo16030173

**Published:** 2026-03-06

**Authors:** Elisa Bientinesi, Alessia Vignoli, Sara Ristori, Maria Salobehaj, Gianmarco Bertoni, Daniela Monti, Leonardo Tenori

**Affiliations:** 1 Department of Experimental and Clinical Biomedical Sciences “Mario Serio”, University of Florence, 50134 Florence, Italy; bientinesi.e@gmail.com (E.B.); sara.ristori@unifi.it (S.R.); gianmarco.bertoni@unifi.it (G.B.); 2 Department of Chemistry “Ugo Schiff”, University of Florence, 50019 Sesto Fiorentino, Italy; alessia.vignoli@unifi.it (A.V.); maria.salobehaj@unifi.it (M.S.); 3 Magnetic Resonance Center (CERM), University of Florence, 50019 Sesto Fiorentino, Italy

**Keywords:** astrocytes, Alzheimer’s disease, exo-metabolome, endo-metabolome, NMR, amyloid β-Protein

## Abstract

**Background/Objectives**: Astrocytes play a critical role in maintaining brain homeostasis and are increasingly recognized as active contributors to neurodegenerative processes. Metabolic dysfunction in astrocytes has been implicated in the onset and progression of Alzheimer’s disease (AD), yet the underlying metabolic alterations remain poorly characterized. **Methods**: We used an optimized protocol for untargeted metabolomic profiling of both intracellular and extracellular compartments of primary human astrocytes derived from AD patients and healthy subjects (HS) using ^1^H nuclear magnetic resonance (NMR) spectroscopy. Cells were treated with oligomeric Aβ_1-42_ to model pathological conditions. **Results**: Aβ_1-42_ treatment induced intracellular metabolic alterations in both AD and HS astrocytes, including a consistent reduction in phosphocreatine, potentially indicating impaired energy-buffering capacity. Notably, a decrease in β-alanine was observed only in AD astrocytes, suggesting alterations in carnosine-related antioxidant defence. Analysis of conditioned media revealed differential responses between groups: AD astrocytes showed increased extracellular levels of 2-oxoglutarate, citrate, and glycine, whereas HS astrocytes exhibited reduced extracellular levels of leucine and isoleucine, suggesting distinct adaptive metabolic responses to Aβ-induced stress. However, none of these differences remained statistically significant after correction for multiple testing. **Conclusions**: These findings suggest that NMR-based metabolomics can detect subtle metabolic shifts in human astrocyte models of AD and HS exposed to amiloidogenic challenge. Given the limited sample size and the exploratory design adopted, the results should be interpreted as preliminary and require validation in larger, better-matched cohorts. Nevertheless, this study provides a methodological framework and generates biologically plausible hypotheses regarding astrocyte metabolic responses relevant to AD pathophysiology.

## 1. Introduction

Astrocytes are emerging as metabolically dynamic cells that play essential roles in maintaining brain homeostasis. As the most abundant glial cell type in the central nervous system, astrocytes actively participate in the uptake, storage, and redistribution of metabolic substrates such as glucose, amino acids, and lipids, supporting both their own cellular functions and those of the surrounding neural environment [1]. Their metabolic flexibility enables them to respond to changes in energy demand, oxidative stress, and nutrient availability, thereby contributing to neuroprotection, gliotransmission, and the modulation of inflammation [2,3,4]. In addition to providing energy and supporting essential biosynthetic and detoxification processes, astrocytes play a central role in coordinating various metabolic processes, including glycolysis, the tricarboxylic acid (TCA) cycle, glutamate–glutamine cycling, and lipid metabolism [5,6]. This intricate metabolic network is crucial not only for maintaining astrocyte viability and function but also for adapting to pathological insults. Accordingly, astrocyte metabolism is increasingly investigated in the context of ageing and neurodegenerative disorders, where alterations in metabolic profiles may reflect disease-associated phenotypes or compensatory mechanisms [7].

Alzheimer’s disease is the leading cause of dementia in elderly people and represents a growing challenge for healthcare systems worldwide. It is clinically characterized by progressive cognitive decline and neuropathologically by brain atrophy, synapse and neuronal loss, chronic neuroinflammation, and the accumulation of two hallmark lesions: extracellular Aβ plaques and intracellular neurofibrillary tangles composed of hyperphosphorylated Tau protein [8]. While Tau aggregation in neurofibrillary tangles within neurons contributes to some aspects of the disease, current evidence supports amyloid plaques as a central pathogenic mechanism, in which misfolded Aβ peptides, particularly in their oligomeric, soluble forms, play a crucial neurotoxic role [9,10]. Aβ oligomers, derived from the sequential proteolysis of the amyloid precursor protein, can disrupt synaptic function, promote oxidative stress, and trigger inflammatory responses in surrounding cells [11,12].

Although neuronal degeneration is a defining feature of AD, increasing attention has focused on the contribution of glial cells, particularly astrocytes, to disease onset and progression. Therefore, studying metabolic changes in astrocytes exposed to molecular stressors, such as Aβ peptides, is crucial to understanding AD pathophysiology and may reveal novel therapeutic targets.

Metabolomics, a discipline dedicated to the identification, quantification, and characterization of the entire spectrum of endogenous and exogenous metabolites in a biological specimen [13,14,15], can facilitate characterization of astrocyte metabolic changes, providing insights into AD progression and identifying potential biomarkers.

The rationale for Aβ_1-42_ treatment was to reproduce a key amyloidogenic stressor in Alzheimer’s disease and to investigate astrocyte metabolic responses to Aβ oligomers. Soluble Aβ_1-42_ species are considered among the most neurotoxic and biologically active forms of Aβ and are known to directly impair astrocytic energy metabolism, redox balance, and mitochondrial function [9,16,17]. By applying Aβ_1-42_ to primary human astrocytes, we aimed to model early AD-relevant pathological conditions and to disentangle disease-specific metabolic vulnerabilities of AD-derived astrocytes from general stress responses.

In a previous study of the same cohort of human primary astrocytes derived from healthy subjects and AD patients, we investigated the differential induction of astrocyte reactivity and cellular senescence following exposure to Aβ_1-42_ [18]. That work demonstrated that AD-derived astrocytes are more susceptible to amyloid-induced stress via inflammatory and senescence-associated pathways. However, the metabolic mechanisms underlying these phenotypic differences remained unexplored.

The present study addresses this gap by focusing specifically on the endo- and exo-metabolomic profiles of the same astrocyte lines under identical experimental conditions. Rather than re-examining reactivity or senescence markers, here, we investigate whether disease-specific metabolic signatures accompany or potentially underlie the previously observed phenotypic alterations.

**Figure 1:**
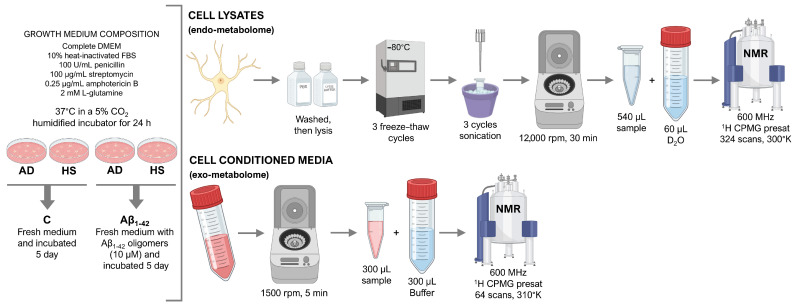
Experimental protocol. A graphical representation of the experimental protocol used for profiling of endo- and exo-metabolomes of human astrocytes by NMR. Created with BioRender.com.

Here, for the first time, to the best of our knowledge, we describe a protocol for endo- (cell lysates) and exo- (cell conditioned media) metabolome analysis of human primary astrocytes, using ^1^H nuclear magnetic resonance (NMR) spectroscopy, including cell culture treatment, sample preparation, acquisition of spectra, and metabolite assignment (Figure 1). Human primary astrocytes derived from both healthy subjects (HS) and AD patients were treated with Aβ_1-42_ oligomers for five days and then investigated by NMR. Metabolite differences were evaluated across four experimental groups: untreated HS cells (HS C), HS cells treated with Aβ_1-42_ (HS Aβ_1-42_), untreated AD cells (AD C), and AD cells treated with Aβ_1-42_ (AD Aβ_1-42_).

## 2. Materials and Methods

### 2.1. Primary Human Astrocyte Culture

Authenticated human primary astrocytes (#36058-01, Celprogen Inc., Torrance, CA, USA) were obtained from six healthy donors (4 females, lot #1514431, #1514413, #1514423, #1514438, and 2 males, lot #1514449, #1514420, with a mean age of 49 ± 4.9 years, age range: 33–65 years) and from five AD patients (2 females, lot #20274, #20262, and 3 males, lot #20255, #20256, #20260, with a mean age of 83 ± 5.6 years, age range: 73–88 years). The supplier provided characterization for astrocyte markers only; other donor metadata, including post-mortem interval, comorbidities, medications, ApoE, Braak stage, etc., were not available.

Cells were grown in complete Dulbecco’s Modified Eagle Medium (glucose concentration 4500 mg/L) (#ECB7501L, Euroclone, Milan, Italy) supplemented with 10% heat-inactivated fetal bovine serum (FBS) (#ECS5000L, Euroclone, Milan, Italy), 100 U/mL penicillin, 100 µg/mL streptomycin, 0.25 µg/mL amphotericin B (#ECM0010D, Euroclone, Milan, Italy), and 2 mM L-glutamine (#ECB3000D, Euroclone, Milan, Italy), and maintained at 37 °C in a 5% CO_2_ humidified incubator.

### 2.2. Preparation of Aβ_1-42_ Oligomers and Astrocyte Treatment

Astrocytes were exposed to oligomeric Aβ_1-42_ to simulate amyloid-related stress relevant to Alzheimer’s disease (AD) pathogenesis. Amyloid β-Protein (1-42) (#4014447; Bachem, Bubendorf, Switzerland) was prepared and oligomerized as previously described [19]. Briefly, the lyophilized peptide was dissolved in 1,1,1,3,3,3-hexafluoro-2-propanol (#105228, Sigma-Aldrich, St. Louis, MO, USA) to 1 mM concentration to obtain monomeric Aβ_1-42_, aliquoted, and stored at −20 °C until use. To generate oligomeric Aβ_1-42_, the solvent was evaporated, and the peptide film was resuspended in dimethyl sulfoxide (D8418, Sigma-Aldrich, St. Louis, MO, USA) at a final peptide concentration of 5 mM. The solution was sonicated in an ultrasonic bath sonicator (USC100T, Avantor^®^, VWR International, Radnor, PA, USA) for 10 min to promote oligomer formation. The suspension was then diluted in complete culture medium supplemented with 2% FBS to a final concentration of 10 µM (monomer equivalent) and incubated at 4 °C for 24 h.

Primary human astrocytes (5.3 × 10^3^ cells/cm^2^) were plated for 24 h in complete medium. The medium was then replaced, and cells were treated with Aβ_1-42_ oligomers (10 µM) starting on day 1 and maintained under the same conditions for 5 days. The selected concentration (10 µM, monomer equivalent) and exposure time (5 days) were based on our previous research [18], which showed that these conditions induce a senescence-associated, pro-inflammatory phenotype in this astrocyte model, and demonstrated efficient Aβ_1-42_ uptake using confocal microscopy and biochemical techniques [18]. The astrocyte lines used in the present study are the same as those previously characterized for reactivity and senescence markers. However, the metabolomic analyses presented here were generated independently and were not included in the previous publication.

Each donor was considered an independent biological replicate. For each donor, parallel control and Aβ_1-42_ treated samples were generated. Statistical analyses were performed using donor-level data (HS: n = 6; AD: n = 5 per condition).

### 2.3. Collection of Culture-Conditioned Medium and Cell Lysates for Metabolomic Analysis

At the end of the experiment, after 5 days of Aβ_1-42_ treatment or no treatment for controls, the conditioned medium was collected, centrifuged at 1500 rpm for 5 min to remove cellular debris, and immediately stored at −80 °C for exo-metabolome analysis. For cell lysate collection, cells were first washed with 1X PBS, then the lysis buffer was added, and cells were detached using a cell scraper (trypsin was avoided). The lysis buffer consisted of PBS supplemented with protease inhibitors: sodium orthovanadate and Halt™ Protease Inhibitor Cocktail EDTA-Free (#78425, ThermoFisher Scientific, Waltham, MA, USA), both diluted 1:2000. The volume of lysis buffer was adjusted to ensure a final lysate volume of at least 600 µL. The lysate from each donor culture was collected into a single tube and subjected to three freeze–thaw cycles at −80 °C to promote cell disruption. Subsequently, lysates were sonicated on ice using the sonicator (#FB120, ThermoFisher Scientific, Waltham, MA, USA) with the following parameters: amplitude 50%; pulsing 15 s on and 15 s off, for a total of 5 cycles (cumulative sonication time 1 min and 15 s). After sonication, lysates were centrifuged at 12,000 rpm for 30 min at 4 °C, and the supernatant was collected, transferred to a new tube, and immediately stored at −80 °C for endo-metabolome analysis.

### 2.4. NMR Sample Preparation

This protocol is an adaptation and fine-tuning of a previously described protocol for ovarian cancer cells [20]. Frozen astrocyte lysates were thawed at room temperature. For each sample, a total of 60 μL of deuterated water (D_2_O), containing 0.58 mM 3-(Trimethylsilyl)propionate-2,2,3,3-d4 as NMR reference, was added to 540 μL of cell lysate, and the mixture was transferred into a 5 mm NMR tube. Frozen cell conditioned media were thawed at room temperature. A total of 300 μL of 75 mM Na_2_HPO_4_ buffer, 20% (*v*/*v*) D_2_O, 4.6 mM 3-(Trimethylsilyl)propionate-2,2,3,3-d4, 6.1 mM NaN_3_, pH 7.4, was added to 300 μL of cell medium, and the mixture was transferred into a 5 mm NMR tube.

### 2.5. NMR Analysis

^1^H NMR spectra were acquired using a Bruker 600 MHz spectrometer (Bruker BioSpin GmbH, Ettlingen, Germany) operating at 600.13 MHz proton Larmor frequency equipped with a 5 mm PATXI ^1^H-^13^C-^15^N and ^2^H-decoupling probe including a *z*-axis gradient coil, an automatic tuning–matching (ATM) system and an automatic refrigerated (6 °C) sample changer (SampleJet, Bruker BioSpin). To ensure high spectral quality and reproducibility, the spectrometer was calibrated daily in accordance with strict standard operating procedures (including solvent suppression and temperature control).

The spectra of cell lysates were acquired at 300 K using the Carr–Purcell–Meiboom–Gill (CPMG) one-dimensional spin–echo sequence (Bruker sequence cpmgpr1d) [21] with water presaturation, 348 scans, 73,728 data points, a spectral width of 12,019 Hz, a relaxation delay of 6 s, and a total spin-echo delay of 80 ms.

The spectra of the cell conditioned media were acquired at 310 K with a 1D CPMG pulse sequence (Bruker sequence cpmgpr1d) with water presaturation, 64 scans, 73,728 data points, a spectral width of 12,019 Hz, a relaxation delay of 4 s, and a total spin-echo delay of 80 ms.

Before applying the Fourier transform, free induction decays were multiplied by an exponential function equivalent to a 0.3 Hz line-broadening factor. Transformed spectra were automatically corrected for phase and baseline distortions and calibrated to the anomeric glucose doublet at δ 5.24 ppm. NMR signals of metabolites were manually assigned using the Chenomx NMR Suite 12, freely available databases, and published literature. The identified metabolites were quantified (in arbitrary units) by integrating the NMR regions of interest using an in-house R script. A panel of 40 and 33 metabolites were quantified in cell lysates and cell conditioned media, respectively (Table 1). The metabolites quantified in the conditioned media included both molecules secreted by the cells (which were absent in the fresh medium) and molecules produced or consumed by the cells that were already present in the fresh medium.

### 2.6. Statistical Analysis

All statistical analyses were performed in the R environment (version 4.5.1). The matrices of quantified metabolites in cell lysates and cell conditioned media were processed separately using the Harmony algorithm [22] to reduce technical noise arising from minor day-to-day differences in cell growth and culture handling. Notably, batch labels (i.e., days of experiments) showed no association with the biological groups under investigation (i.e., treated vs. untreated in AD and HS). Harmonized data were scaled without centering.

The Student’s *t*-test was performed in order to explore differences in metabolites between the groups of interest (HS Aβ_1-42_ vs. HS C, and AD Aβ_1-42_ vs. AD C). The assumptions of the Student’s *t*-test were assessed using the Shapiro–Wilk test for normality; metabolites with non-normal distributions were analyzed using the Wilcoxon test. The Welch approximation to the degrees of freedom was used as the default setting in the R function “t.test”. All *p*-values were also adjusted for multiple testing using the false discovery rate (FDR) procedure with Benjamini–Hochberg correction at α = 0.05. Cliff’s delta effect size of each metabolite was calculated using the R function “cliff.delta”, package “effsize”. To assess whether donor age acted as a potential confounding factor, Pearson’s correlation analyses were conducted between donor age and the concentrations of all quantified metabolites in untreated cells for both cell lysates and conditioned media.

## 3. Results

In this study, we used NMR spectroscopy to obtain a comprehensive, high-throughput, and simultaneous analysis of endo- (cell lysates) and exo-metabolomes (cell conditioned media) from primary human astrocyte cultures of AD patients and healthy subjects, either untreated or treated with Aβ_1-42_ oligomers.

**Figure 2:**
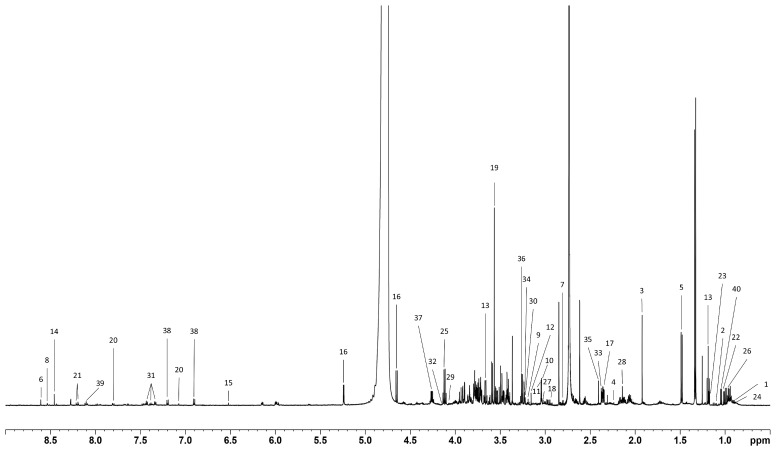
A representative ^1^H NMR CPMG spectrum of the endo-metabolome (cell lysate) with metabolite assignment: 1. 2-oxoisocaproate, 2. 3-methyl-2-oxovalerate, 3. Acetate, 4. Acetone, 5. Alanine. 6. AMP, 7. Aspartate, 8. ATP, 9. β-alanine, 10. Creatine, 11. Creatine-phosphate, 12. Dimethyl sulfone, 13. Ethanol, 14. Formate, 15. Fumarate, 16. Glucose, 17. Glutamate, 18. Glutathione, 19. Glycine, 20. Histidine, 21. Hypoxanthine, 22. Isoleucine, 23. Isopropanol, 24. Isovalerate, 25. Lactate, 26. Leucine, 27. Lysine, 28. Methionine, 29. Myo-inositol, 30. O-acetylcholine, 31. Phenylalanine, 32. Proline, 33. Pyruvate, 34. Sn-glycerol-3-phosphocholine, 35. Succinate, 36. Taurine, 37. Threonine, 38. Tyrosine, 39. UMP, 40. Valine.

**Figure 3:**
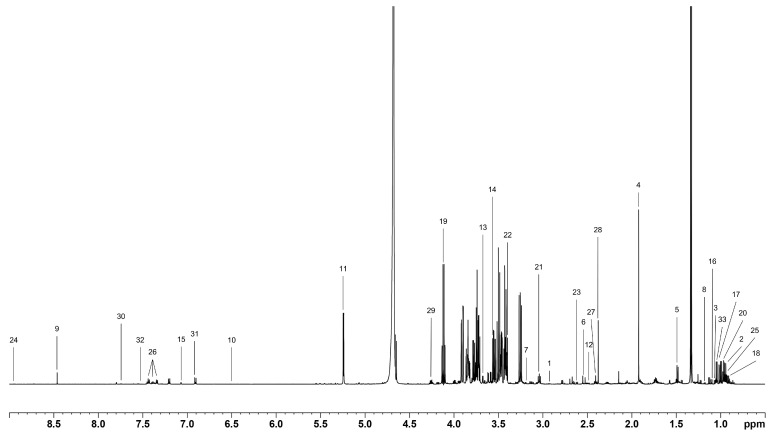
A representative ^1^H NMR CPMG spectrum of the exo-metabolome (cell conditioned media) with metabolite assignment: 1. 2-oxoglutarate, 2. 2-oxoisocaproate, 3. 3-methyl-2-oxovalerate, 4. Acetate, 5. Alanine, 6. Citrate, 7. Cystine, 8. Ethanol, 9. Formate, 10. Fumarate, 11. Glucose, 12. Glutamine, 13. Glycerol + Myo-Inositol, 14. Glycine, 15. Histidine, 16. Isobutyrate, 17. Isoleucine, 18. Isovalerate, 19. Lactate, 20. Leucine, 21. Lysine, 22. Methanol, 23. Methionine, 24. Niacinamide, 25. Pantothenate, 26. Phenylalanine, 27. Pyroglutamate, 28. Pyruvate, 29. Threonine, 30. Tryptophan, 31. Tyrosine, 32. Uracil, 33. Valine.

As described in the Methods section, two panels of 40 and 33 metabolites were identified and quantified in cell lysates and cell conditioned media, respectively (Figure 2 and Figure 3).

Given the exploratory nature of this proof-of-concept study and the limited sample size, statistical significance in the Results section refers to uncorrected *p*-values (*p* < 0.05). None of the observed differences remained statistically significant after Benjamini–Hochberg false discovery rate correction. Accordingly, the findings should be interpreted as hypothesis-generating. Nevertheless, the data provide important methodological insights and demonstrate the feasibility and sensitivity of NMR-based metabolomics for the integrated analysis of intracellular and extracellular metabolic profiles in human astrocyte models.

NMR analysis enabled the identification of multiple metabolites and the assessment of their changes under the experimental conditions. Intracellular and extracellular metabolomic profiles showed partially overlapping but differential responses to Aβ_1-42_ treatment, highlighting compartment-specific metabolic adaptations in astrocytes.

**Figure 4:**
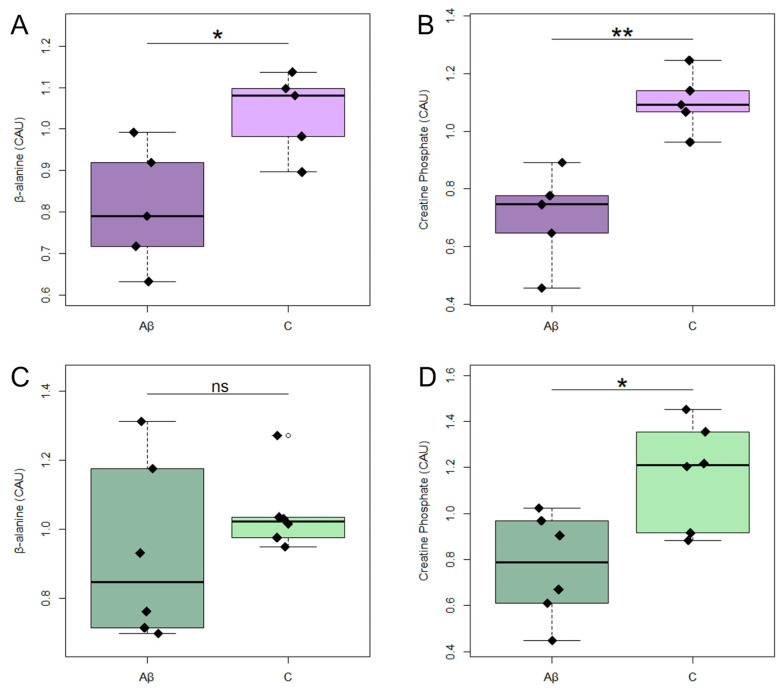
NMR-determined relative concentrations of altered metabolites in cell lysates (endo-metabolome) of astrocytes in untreated cells (C, light colours) or treated with Aβ_1-42_ oligomers (Aβ, dark colours). In astrocytes from AD: (**A**) β-alanine; (**B**) creatine phosphate. In astrocytes of HS: (**C**) β-alanine; (**D**) creatine phosphate. None of these metabolites remains significant after FDR correction. Cell lysates were collected after 5 days of Aβ_1-42_ oligomer treatment. * *p*  <  0.05; ** < 0.01; ns *p* > 0.05. Abbreviations: C: untreated cells; Aβ: treated with Aβ_1-42_ oligomers; AD: astrocytes from Alzheimer’s patients; HS: astrocytes from healthy subjects.

**Figure 5:**
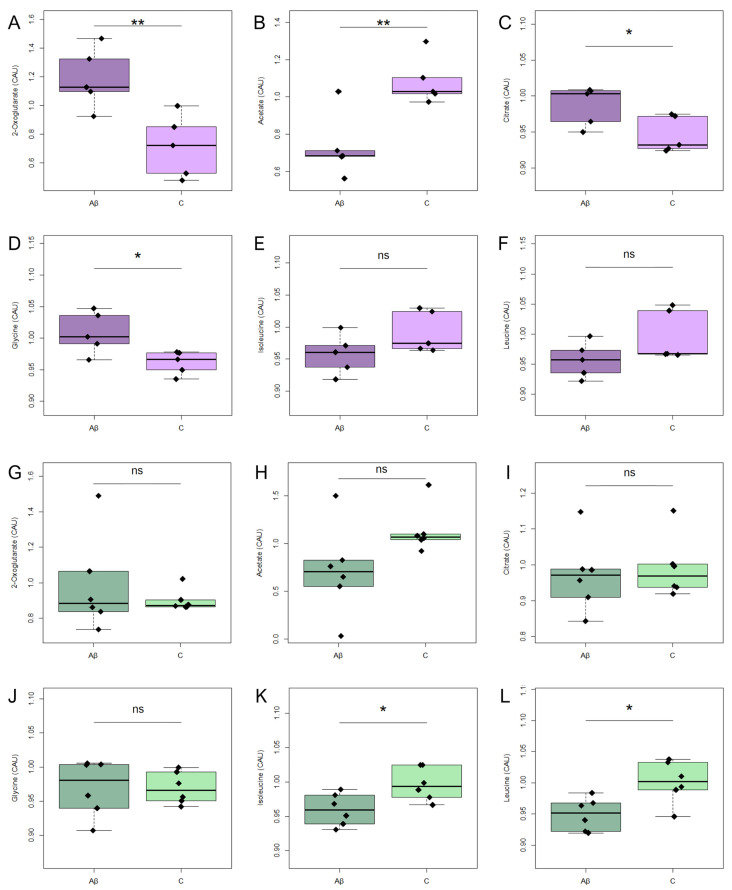
NMR-determined relative concentrations of altered metabolites in astrocyte conditioned media (exo-metabolome) from untreated cells (C, light colours) or cells treated with Aβ_1-42_ oligomers (Aβ, dark colours). In astrocyte media from AD: (**A**) 2-oxoglutarate; (**B**) acetate; (**C**) citrate; (**D**) glycine; (**E**) isoleucine; (**F**) leucine. In astrocyte media from C: (**G**) 2-oxoglutarate; (**H**) acetate; (**I**) citrate; (**J**) glycine; (**K**) isoleucine; (**L**) leucine. None of these metabolites remain significant after FDR correction. Conditioned media were collected after 5 days of Aβ_1-42_ oligomer treatment. * *p*  <  0.05; ** < 0.01; ns *p* > 0.05. Abbreviations: C: untreated cells; Aβ: treated with Aβ_1-42_ oligomers; AD: astrocytes from Alzheimer’s patients; HS: astrocytes from healthy subjects.

Treatment with Aβ_1-42_ oligomers significantly alters the intracellular levels (endo-metabolome) of creatine phosphate and β-alanine (Figure 4A,B), as well as the extracellular levels (exo-metabolome) of 2-oxoglutarate, acetate, citrate and glycine, in cell cultures derived from AD patients (Figure 5A–D). Treatment with Aβ_1-42_ significantly altered intracellular levels of creatine phosphate (Figure 4D), but not those of alanine (Figure 4C), in HS-derived astrocytes. The extracellular levels of isoleucine and leucine in HS-derived astrocytes were significantly altered by treatment with Aβ_1-42_ (Figure 5K,L). However, none of these alterations survived FDR correction.

No significant correlations were observed between donor age and the concentrations of metabolites significantly modulated by Aβ exposure (Supplementary Tables S1 and S2).

For the sake of completeness, the full trends of metabolites in cell lysates and conditioned media in untreated cells and cells treated with Aβ_1-42_ oligomers are provided as Supplementary Tables S3 and S4.

## 4. Discussion

Astrocytes play a key role in brain metabolism and homeostasis [23]. In this study, we present a protocol for metabolomic profiling of astrocyte cell lysates and culture media using NMR spectroscopy to characterize the metabolic responses to Aβ_1-42_ oligomer treatment in astrocytes derived from both AD patients and HS.

Our current findings build on earlier research with the same cohort of human primary astrocytes, showing that exposure to Aβ_1-42_ triggers distinct pro-inflammatory and senescence-related responses in AD-derived astrocytes compared with healthy astrocytes [18]. While the previous study focused on phenotypic markers of astrocyte reactivity and aging, the underlying metabolic basis for this differential susceptibility was not investigated. Given the tight interplay among cellular metabolism, inflammatory activation, and senescence programmes, we hypothesized that amyloid-induced phenotypic changes may be accompanied by disease-specific metabolic remodelling. The metabolomic alterations described here may therefore provide complementary insights, suggesting that the previously observed differences in stress susceptibility are paralleled by distinct shifts in energy buffering, mitochondrial intermediates, and amino acid handling.

Treatment with Aβ_1-42_ was associated with alterations in the endo-metabolome, although these differences did not remain significant after multiple testing correction. Although these results should be interpreted with caution, both AD- and HS-derived astrocytes showed reduced intracellular phosphocreatine levels (Figure 4B,D). Creatine phosphate serves as a temporary buffer of high-energy phosphate groups, regenerating ATP from accumulating adenosine diphosphate when ATP regeneration by glycolysis or mitochondrial metabolism is insufficient [24]. Its consistent depletion suggests an impaired energy-buffering capacity under stress, a feature associated with mitochondrial dysfunction, metabolic stress and compromised ATP regeneration, especially in AD [25]. Interestingly, intracellular β-alanine levels were significantly reduced in Aβ_1-42_ treated AD astrocyte cultures, potentially suggesting a differential metabolic response in AD astrocytes (Figure 4A,C). Given that β-alanine is a rate-limiting precursor for carnosine synthesis, this reduction may reflect an impaired ability to sustain carnosine-mediated antioxidant defence [26]. Carnosine is a dipeptide with well-established roles in buffering oxidative stress and protecting cellular components from damage [27,28,29]. This interpretation is supported by previous studies demonstrating that carnosine exerts neuroprotective effects in astrocytes by mitigating oxidative stress, modulating inflammatory responses, and preserving mitochondrial function [29,30,31]. These combined actions suggest that diminished β-alanine availability, as a precursor of carnosine, could further exacerbate oxidative imbalance and neuroinflammation in AD cells.

Notably, analysis of conditioned media after Aβ_1-42_ exposure elicited different metabolic responses in AD and HS astrocyte cultures, particularly involving metabolites related to the TCA cycle and amino acid metabolism. In AD astrocytes, the treatment led to significant increases in extracellular 2-oxoglutarate, citrate, and glycine (Figure 5A,C,D). These alterations may reflect differential modulation of mitochondrial and amino acid pathways under amyloidogenic stress [32,33]. Specifically, extracellular acetate levels decreased in both AD and HS cultures, but the reduction was statistically significant only in AD (Figure 4B). This observation aligns with previous evidence linking altered astrocytic acetate metabolism to neuroinflammation and AD-related metabolic dysfunction [34,35]. The reduction in acetate may reflect increased intracellular utilization for acetyl-Coenzyme A synthesis or impaired efflux, consistent with disrupted energy substrate handling in stressed astrocytes [1,36]. Overall, the selective increase in glycine and TCA cycle intermediates observed in AD astrocytes may reflect impaired mitochondrial activity or a dysregulated anaplerotic response, the metabolic process that replenishes TCA cycle intermediates.

In this context, HS astrocyte conditioned media showed a non-significant increase in 2-oxoglutarate levels after treatment (Figure 5G); however, there was a significant reduction in leucine and isoleucine levels (Figure 5K,L). These essential amino acids are known to serve as alternative energy substrates in the brain, particularly in astrocytes and neurons, through their catabolism into acetyl-CoA and succinyl-CoA, intermediates of the TCA cycle [37]. In addition to their role in energy metabolism, leucine and isoleucine contribute to neurotransmitter homeostasis and nitrogen balance by participating in glutamate synthesis and transamination reactions [38]. Leucine, in particular, is a potent activator of the mammalian Target of Rapamycin signalling pathway, which regulates protein synthesis and cellular growth [39]. The observed decrease in leucine and isoleucine in HS astrocytes may indicate increased cellular uptake as a compensatory response to sustain energy production and biosynthesis. However, in Aβ_1-42_ treated HS astrocytes, this depletion likely reflects impaired metabolic adaptation to stress, potentially disrupting bioenergetic support and astrocyte–neuron metabolic coupling [5].

A key strength of this study lies in the combined analysis of intracellular (endo-) and extracellular (exo-) metabolomes, which provide complementary but non-redundant information on astrocyte metabolic responses to Aβ_1-42_ exposure [13,21,40]. Changes observed in the endo-metabolome primarily reflect alterations in intracellular energy homeostasis, redox balance, and mitochondrial function, processes in which astrocytes play a central role in the brain [5,6,41]. In contrast, variations in the exo-metabolome capture the net outcome of metabolite uptake, secretion, and metabolic exchange with the extracellular environment, providing a metabolic footprint of astrocyte functional activity and substrate handling. The observation that certain alterations, such as phosphocreatine depletion, were confined to the intracellular compartment suggests a direct impairment of energy-buffering mechanisms that is not immediately mirrored in the extracellular compartment, consistent with astrocytic mitochondrial stress responses [24,32]. Conversely, changes in the concentrations of TCA cycle intermediates and amino acids in conditioned media indicate active metabolic remodelling and altered substrate utilization, which may reflect compensatory or maladaptive astrocyte responses to amyloid-induced stress [33,37,38]. Metabolic alterations in the endo- and exo-metabolomes underscore the importance of simultaneously profiling both compartments, as similar trends point to generalized stress responses, whereas divergent patterns reveal compartment-specific regulation.

The limitations of our study should be acknowledged. First, the relatively small sample size (n = 6 healthy donors, n = 5 AD donors) limits statistical power and the ability to detect subtle metabolic differences. Second, the substantial age difference between healthy and AD donors could represent a potential confounding factor; however, our analyses do not support a major age-driven effect on the metabolites of interest. A further limitation is that donor metadata beyond astrocyte marker characterization were not available (e.g., post-mortem interval, comorbidities, medications, ApoE genotype, Braak stage), which does not allow us to explore correlations between these variables and the observed metabolic profiles. Finally, while our study provides detailed profiling of intracellular and extracellular metabolites, mechanistic interpretations remain speculative and require validation in larger, well-matched cohorts. Despite these limitations, the results highlight the potential of NMR metabolomics to identify subtle metabolic alterations in astrocytes relevant to AD.

## 5. Conclusions

Overall, the metabolomic profiles revealed both common and divergent responses to Aβ_1-42_ exposure in AD and HS astrocytes. Shared alterations, such as creatine phosphate depletion, likely reflect general stress-related metabolic mechanisms, whereas differences in TCA cycle intermediates and amino acid metabolism may indicate differences in response magnitude or cellular adaptability to Aβ_1-42_-induced toxicity. Although the metabolic shifts were modest, their biological relevance is underscored by the literature linking these pathways to neurodegenerative processes. These findings highlight the utility of NMR-based metabolomics for uncovering subtle but biologically meaningful metabolic changes in astrocyte models. As a non-destructive, quantitative, and reproducible technique, NMR is especially valuable for characterizing complex metabolic phenotypes in neurodegenerative disease research.

In conclusion, our exploratory findings suggest differential metabolic responses to Aβ_1-42_ exposure in AD and HS astrocytes, highlighting both shared stress-related alterations and potentially divergent adaptive patterns. Although these metabolic shifts did not remain statistically significant after multiple testing correction and require validation in larger, well-matched cohorts, they generate biologically plausible hypotheses about astrocyte metabolic dynamics in AD. These data further support the utility of NMR-based metabolomics as a robust platform for investigating astrocyte-specific metabolic phenotypes in neurodegenerative research.

## Figures and Tables

**Table 1:** ^1^H chemical shifts of assigned metabolites in 1D CPMG spectra of cell lysates (L) and cell conditioned media (CM). Multiplicity is indicated as follows: s: singlet, d: doublet, t: triplet, q: quartet, m: multiplet.

Metabolite	^1^H Chemical Shift (ppm)	Multiplicity	Biosample
Pantothenate	0.90	s	CM
Isovalerate	0.92	d	L, CM
2-oxoisocaproate	0.94	d	L, CM
Leucine	0.96	m	L, CM
Isoleucine	1.02	d	L, CM
Valine	1.04	d	L, CM
Isobutyrate	1.07	d	CM
3-methyl-2-oxovalerate	1.11	d	L, CM
Isopropanol	1.18	d	L
Ethanol	1.20	t	L, CM
Alanine	1.49	d	L, CM
Acetate	1.92	s	L, CM
Methionine	2.14	s	L, CM
Acetone	2.24	s	L
Glutamate	2.37	m	L
Pyruvate	2.38	s	L, CM
Pyroglutamate	2.40	m	CM
Succinate	2.40	s	L
Glutamine	2.45	m	CM
Citrate	2.53	d	CM
Aspartate	2.68	m	L
Glutathione	2.95	m	L
2-oxoglutarate	3.01	t	CM
Creatine	3.04	s	L
Lysine	3.04	t	L, CM
Creatine-phosphate	3.05	s	L
Dimethyl sulfone	3.16	s	L
Cystine	3.17	q	CM
β-alanine	3.19	t	L
O-acetylcholine	3.22	s	L
Sn-glycerol-3-phosphocholine	3.23	s	L
Taurine	3.26	t	L
Methanol	3.37	s	CM
Glycine	3.56	s	L, CM
Glycerol + Myo-Inositol	3.66	q	CM
Myo-Inositol	4.07	t	L
Lactate	4.12	m	L, CM
Proline	4.14	m	L
Threonine	4.26	m	L, CM
Glucose	5.24	d	L, CM
Fumarate	6.52	s	L, CM
Tyrosine	6.90	m	L, CM
Histidine	7.07	s	L, CM
Phenylalanine	7.33	m	L, CM
Uracil	7.54	d	CM
Tryptophan	7.74	m	CM
Uridine monophosphate (UMP)	8.11	d	L
Hypoxanthine	8.21	s	L
Formate	8.46	s	L, CM
Adenosine triphosphate (ATP)	8.53	s	L
Adenosine monophosphate (AMP)	8.60	s	L
Niacinamide	8.94	m	CM

## Data Availability

Raw concentrations of metabolites in cell lysates and cell conditioned media generated from this study are available as Supplementary File.

## Supplementary Materials

The following supporting information can be downloaded at: https://www.mdpi.com/article/10.3390/metabo16030173/s1. Table S1: Pearson correlation analysis between metabolite concentrations in cell lysates and age of donors; Table S2: Pearson correlation analysis between metabolite concentrations in conditioned media and age of donors; Table S3: Comparisons of metabolite concentrations in cell lysates from AD patients and HS; Table S4: Comparisons of metabolite concentrations in conditioned media where astrocytes from AD patients and HS were grown. Excel file named “Manuscript_data” with raw metabolite concentrations in cell lysates and cell conditioned media.

## Abbreviations

The following abbreviations are used in this manuscript:

NMR	Nuclear Magnetic Resonance
AD	Alzheimer’s disease
HS	Healthy subjects
TCA	Tricarboxylic acid
C	Controls
FBS	Fetal bovine serum
Aβ	Amyloid β-Protein
PBS	Phosphate-Buffered Saline
CPMG	Carr–Purcell–Meiboom–Gill
UMP	Uridine monophosphate
ATP	Adenosine triphosphate
AMP	Adenosine monophosphate
CAU	Concentrations measured in arbitrary units
CoA	Coenzyme A
