# Supplementary material for: An NMR-Based Protocol for Profiling the Endo- and Exo-Metabolomes in Aβ1-42 Treated Human Astrocytes from Healthy and Alzheimer’s Disease Donors
Source: Metabolites. 2026 Mar 6;16(3):173. doi: 10.3390/metabo16030173 (PMC13027727; doi:10.3390/metabo16030173)
Supplement: Supplementary file 1 [file metabolites-16-00173-s001.zip › Supplementary Materials.pdf]

Supplementary Table S1. Pearson correlation analysis between metabolite concentrations in cell lysates and age of donors. This analysis was performed for untreated cells only.

	**Correlation coefficient**	**p-value**
Glutathione	-0.519	0.102
sn-Glycerol-3-phosphocholine	0.478	0.137
O-Acetylcholine	0.432	0.184
Acetate	-0.399	0.224
Alanine	-0.397	0.227
Isopropanol	0.374	0.258
Glycine	0.337	0.310
Lysine	-0.328	0.325
Formate	0.325	0.330
Succinate	-0.324	0.331
Threonine	-0.322	0.335
AMP	-0.318	0.340
Hypoxanthine	-0.302	0.367
Creatine Phosphate	-0.301	0.368
Dimethyl sulfone	-0.287	0.391
Isovalerate	-0.283	0.398
Phenylalanine	-0.278	0.407
β-alanine	-0.265	0.431
Fumarate	-0.257	0.446
Lactate	-0.240	0.477
UMP	-0.222	0.512
Pyruvate	0.202	0.551
3-Methyl-2-oxovalerate	0.194	0.568
2-Oxoisocaproate	-0.192	0.571
Creatine	-0.191	0.574
Valine	-0.183	0.591
Isoleucine	-0.180	0.596
Leucine	-0.172	0.613
myo-Inositol	0.162	0.633
Histidine	-0.161	0.637
Glutamate	0.143	0.674
Tyrosine	-0.116	0.735
Ethanol	-0.095	0.781
Glucose	-0.046	0.892
Aspartate	-0.046	0.892
Acetone	-0.029	0.933
Proline	0.025	0.941
Methionine	0.015	0.965
ATP	0.009	0.979
Taurine	-0.005	0.989

Supplementary Table S2. Pearson correlation analysis between metabolite concentrations in conditioned media and age of donors. This analysis was performed for untreated cells only.

	Correlation coefficient	p-value
Alanine	-0.678	0.022
Phenylalanine	0.625	0.040
Citrate	-0.598	0.052
Tyrosine	0.563	0.071
Methanol	-0.561	0.073
Glycerol-Myo-inositol	-0.557	0.075
Niacinamide	-0.516	0.104
Glucose	0.516	0.105
Acetate	-0.501	0.117
Lactate	-0.482	0.133
2-Oxoglutarate	-0.473	0.142
Uracil	0.380	0.249
3-Methyl-2-oxovalerate	0.378	0.252
2-Oxoisocaproate	0.348	0.295
Pyroglutamate	0.328	0.324
Isobutyrate	-0.310	0.353
Isovalerate	-0.255	0.449
Pyruvate	0.239	0.480
Fumarate	-0.234	0.488
Threonine	0.232	0.492
Histidine	0.223	0.509
Formate	0.214	0.527
Tryptophan	0.204	0.548
Leucine	0.200	0.555
Valine	0.160	0.638
Isoleucine	0.112	0.743
Ethanol	-0.101	0.768
Glutamine	0.094	0.783
Pantothenate	0.078	0.820
Lysine	-0.053	0.877
Cystine	0.049	0.887
Glycine	-0.026	0.938
Methionine	-0.014	0.968

Supplementary Table S3. Comparisons of metabolite concentrations in cell lysates from AD patients and HS. Values are reported as Cliff's delta effect size with 95% confidence interval (CI), p-value, and false discovery rate (FDR)–adjusted p-value.

	AD patients			Healthy controls		
**Metabolite**	**Effect size (95% CI)**	**p-value**	**FDR p-value**	**Effect size (95% CI)**	**p-value**	**FDR p-value**
2-Oxoisocaproate	0.16 (-0.48–0.69)	0.943	0.996	-0.44 (-0.87–0.36)	0.290	0.976
3-Methyl-2-oxovalerate	-0.36 (-0.84–0.44)	0.496	0.996	0.11 (-0.56–0.70)	0.627	0.976
Acetate	-0.52 (-0.90–0.33)	0.153	0.992	-0.50 (-0.87–0.23)	0.184	0.976
Acetone	0.20 (-0.66–0.83)	0.248	0.992	-0.11 (-0.72–0.59)	0.774	0.976
Alanine	0.04 (-0.56–0.61)	0.998	0.998	0.00 (-0.62–0.62)	0.934	0.976
AMP	0.12 (-0.52–0.67)	0.740	0.996	0.17 (-0.43–0.66)	0.939	0.976
Aspartate	0.04 (-0.66–0.70)	0.911	0.996	0.11 (-0.47–0.62)	0.696	0.976
ATP	-0.28 (-0.77–0.42)	0.234	0.992	-0.44 (-0.85–0.29)	0.462	0.976
β-alanine	-0.76 (-0.96–-0.07)	0.023	0.454	-0.39 (-0.86–0.45)	0.357	0.976
Creatine	-0.28 (-0.82–0.53)	0.485	0.996	0.22 (-0.48–0.75)	0.824	0.976
Creatine Phosphate	-1.00 (-1.00–-0.82)	0.003	0.108	-0.72 (-0.96–0.17)	0.013	0.514
Dymethyl sulfone	-0.20 (-0.78–0.57)	0.565	0.996	-0.67 (-0.94–0.11)	0.081	0.976
Ethanol	-0.20 (-0.80–0.60)	0.644	0.996	-0.22 (-0.68–0.37)	0.710	0.976
Formate	-0.60 (-0.92–0.20)	0.151	1.000	0.00 (-0.55–0.55)	1.000	1.000
Fumarate	-0.20 (-0.68–0.41)	0.745	0.996	-0.17 (-0.63–0.39)	0.699	0.976
Glucose	-0.04 (-0.63–0.57)	0.847	0.996	-0.11 (-0.68–0.54)	0.664	0.976
Glutamate	-0.28 (-0.78–0.43)	0.359	0.996	-0.06 (-0.60–0.52)	0.938	0.976
Glutathione	-0.12 (-0.76–0.63)	0.582	0.996	-0.39 (-0.82–0.31)	0.383	0.976
Glycine	-0.60 (-0.92–0.20)	0.151	1.000	-0.33 (-0.75–0.28)	0.394	1.000

Histidine	0.04 (-0.66–0.70)	0.892	0.996	-0.17 (-0.68–0.46)	0.814	0.976
Hypoxanthine	-0.68 (-0.94–0.10)	0.067	0.889	-0.72 (-0.96–0.16)	0.074	0.976
Isoleucine	0.12 (-0.63–0.76)	0.818	0.996	-0.06 (-0.56–0.48)	0.915	0.976
Isopropanol	-0.08 (-0.59–0.48)	0.906	1.000	0.14 (-0.41–0.61)	0.732	1.000
Isovalerate	0.20 (-0.57–0.78)	0.350	0.996	0.00 (-0.56–0.56)	0.819	0.976
Lactate	0.12 (-0.63–0.76)	0.670	0.996	0.00 (-0.50–0.50)	0.880	0.976
Leucine	0.04 (-0.66–0.70)	0.842	0.996	-0.06 (-0.56–0.48)	0.791	0.976
Lysine	0.44 (-0.41–0.88)	0.398	0.996	0.06 (-0.44–0.52)	0.754	0.976
Methionine	-0.04 (-0.63–0.57)	0.937	0.996	0.00 (-0.55–0.55)	0.987	0.987
myo-Inositol	0.36 (-0.28–0.78)	0.740	0.996	0.00 (-0.50–0.50)	0.734	0.976
O-Acetylcholine	-0.44 (-0.84–0.27)	0.124	0.992	0.11 (-0.56–0.70)	0.586	0.976
Phenylalanine	0.36 (-0.44–0.84)	0.368	0.996	-0.06 (-0.63–0.56)	0.861	0.976
Proline	0.12 (-0.52–0.67)	0.964	0.996	0.22 (-0.48–0.75)	0.397	0.976
Pyruvate	0.32 (-0.46–0.82)	0.545	0.996	-0.03 (-0.60–0.56)	0.917	0.976
sn-Glycerol-3-phosphocholine	-0.44 (-0.86–0.34)	0.294	0.996	0.00 (-0.56–0.56)	0.795	0.976
Succinate	0.04 (-0.66–0.70)	0.937	0.996	-0.28 (-0.71–0.30)	0.304	0.976
Taurine	-0.52 (-0.90–0.33)	0.181	0.992	-0.61 (-0.92–0.15)	0.169	0.976
Threonine	0.12 (-0.52–0.67)	0.935	0.996	-0.11 (-0.64–0.48)	0.650	0.976
Tyrosine	0.12 (-0.61–0.74)	0.781	0.996	0.06 (-0.44–0.52)	0.952	0.976
UMP	0.36 (-0.51–0.87)	0.490	0.996	0.39 (-0.15–0.75)	0.454	0.976
Valine	0.12 (-0.63–0.76)	0.798	0.996	-0.06 (-0.56–0.48)	0.907	0.976

Supplementary Table S4. Comparisons of metabolite concentrations in conditioned media where astrocytes from AD patients and HS were grown. Values are reported as Cliff's delta effect size with 95% confidence interval (CI), p-value, and false discovery rate (FDR)–adjusted p-value.

	AD patients			Healthy controls		
**Metabolite**	**Effect size (95% CI)**	**p-value**	**FDR p-value**	**Effect size (95% CI)**	**p-value**	**FDR p-value**
2-Oxoglutarate	0.92 (0.52–0.99)	0.016	0.349	-0.06 (-0.72 – 0.66)	0.937	0.937
2-Oxoisocaproate	0.44 (-0.46–0.89)	0.236	0.410	-0.28 (-0.77 – 0.42)	0.578	0.994
3-Methyl-2-oxovalerate	0.52 (-0.33–0.90)	0.116	0.373	0.11 (-0.47 – 0.62)	0.980	0.994
Acetate	-0.84 (-0.98–-0.17)	0.008	0.136	-0.72 (-0.96 – 0.16)	0.097	0.723
Alanine	0.52 (-0.33–0.90)	0.179	0.405	0.22 (-0.52 – 0.77)	0.621	0.994
Citrate	0.68 (-0.10–0.94)	0.041	0.296	-0.22 (-0.75 – 0.48)	0.733	0.994
Cystine	0.60 (-0.20–0.92)	0.147	0.404	0.08 (-0.56 – 0.66)	0.776	0.994
Ethanol	0.04 (-0.66–0.70)	0.778	0.885	-0.06 (-0.67 – 0.60)	0.925	0.994
Formate	0.36 (-0.44–0.84)	0.187	0.405	0.17 (-0.52 – 0.72)	0.618	0.994
Fumarate	-0.04 (-0.63–0.57)	0.609	0.742	-0.56 (-0.90 – 0.22)	0.068	0.723
Glucose	-0.36 (-0.84–0.44)	0.420	0.603	-0.44 (-0.85 – 0.29)	0.645	0.994
Glutamine	-0.20 (-0.72–0.46)	0.628	0.742	-0.28 (-0.79 – 0.46)	0.740	0.994
Glycerol-Myo-inositol	0.44 (-0.37–0.87)	0.297	0.466	-0.11 (-0.70 – 0.56)	0.746	0.994
Glycine	0.76 (-0.09–0.97)	0.030	0.296	0.17 (-0.58 – 0.76)	0.994	0.994
Histidine	0.44 (-0.13–0.79)	0.233	0.410	0.11 (-0.47 – 0.62)	0.837	0.994
Isobutyrate	0.68 (-0.10–0.94)	0.063	0.296	0.44 (-0.39 – 0.88)	0.295	0.994
Isoleucine	-0.60 (-0.92–0.20)	0.124	0.373	-0.67 (-0.94 – 0.10)	0.023	0.374

Isovalerate	0.68 (-0.10–0.94)	0.062	0.296	0.56 (-0.23 – 0.90)	0.270	0.994
Lactate	0.52 (-0.33–0.90)	0.203	0.405	0.44 (-0.29 – 0.85)	0.340	0.994
Leucine	-0.52 (-0.90–0.33)	0.123	0.373	-0.83 (-0.99 – 0.40)	0.015	0.374
Lysine	0.28 (-0.44–0.78)	0.417	0.603	-0.56 (-0.91 – 0.29)	0.186	0.994
Methanol	0.44 (-0.46–0.89)	0.476	0.629	0.11 (-0.55 – 0.69)	0.871	0.994
Methionine	0.36 (-0.51–0.87)	0.630	0.742	-0.22 (-0.75 – 0.48)	0.450	0.994
Niacinamide	0.84 (0.17–0.98)	0.059	0.296	-0.44 (-0.87 – 0.36)	0.227	0.994
Pantothenate	-0.04 (-0.70–0.66)	0.857	0.926	-0.22 (-0.77 – 0.52)	0.482	0.994
Phenylalanine	-0.36 (-0.81–0.36)	0.209	0.405	0.06 (-0.53 – 0.60)	0.911	0.994
Pyroglutamate	-0.04 (-0.60–0.54)	0.913	0.942	0.06 (-0.46 – 0.55)	0.948	0.994
Pyruvate	0.52 (-0.33–0.90)	0.112	0.373	0.11 (-0.56 – 0.70)	0.678	0.994
Threonine	-0.20 (-0.68–0.41)	0.980	0.980	-0.33 (-0.74 – 0.25)	0.319	0.994
Tryptophan	-0.04 (-0.63–0.57)	0.870	0.926	0.11 (-0.47 – 0.62)	0.849	0.994
Tyrosine	-0.44 (-0.84–0.28)	0.197	0.405	-0.28 (-0.78 – 0.45)	0.578	0.994
Uracil	-0.44 (-0.87–0.37)	0.290	0.466	0.22 (-0.52 – 0.77)	0.972	0.994
Valine	-0.12 (-0.63–0.46)	0.444	0.611	-0.67 (-0.93 – 0.05)	0.110	0.723
